# A simple and rapid ESI-LC-MS/MS method for simultaneous screening of doping agents in urine samples

**DOI:** 10.4103/0253-7613.51347

**Published:** 2009-04

**Authors:** I. Madhusudhana Reddy, Alka Beotra, S. Jain, S. Ahi

**Affiliations:** National Dope Testing Laboratory, Ministry of Youth Affairs and Sports, J. N. Stadium, New Delhi, India

**Keywords:** Detection limit, glucocorticosteroids, LC/MS/MS, validation

## Abstract

**Objective:**

The use of performance enhancing substances is banned in sports by the World Anti-Doping Agency (WADA). Though most prohibited substances can be detected by GC/MS, inclusion of corticosteroids and designer drugs has made it essential to detect these critical doping agents on LC/MS/MS due to their better separation and detection.

**Materials and Methods:**

A common extraction procedure for the isolation of acidic, basic and neutral drugs from urine samples was developed. A total of 28 doping drugs were analyzed on API 3200 Triple quadrupole mass spectrometer using C18 column in atmospheric pressure electrospray ionization. The mobile phase composition was a mixture of 1% formic acid and acetonitrile with gradient time period.

**Results:**

The method developed was very sensitive for detection of 28 doping agents. The linearity was performed for each drug and the total recovery percentage ranged from 57 to 114. Limit of detection is found to be 0.5 ng/ml for carboxy finasteride and 1-5 ng/ml for other drugs. The method was successfully used to detect positive urine samples of 3-OH-stanozolol, methyl phenidate, mesocarb, clomiphene metabolite and carboxy finasteride.

**Conclusion:**

The method developed based on controlled pH extraction method and HPLC-mass spectrometry analysis allowed better identification and confirmation of glucocorticosteroids and a few other drugs in different categories. The validated method has been used successfully for testing of 1000 In-competition samples. The method helped in detection of chemically and pharmacologically different banned drugs in urine in a single short run at a minimum required performance limit set by WADA.

## Introduction

The use and abuse of performance-enhancing substances has been an issue in sports since ancient times. The availability of numerous synthetic steroids and peptide hormones has made testing an analytical challenge.[1] Acknowledging the facts of performance enhancing and deleterious side effects, the World Anti-doping Agency (WADA) banned these drugs in sports.[2] The techniques of dope testing have improved immensely from 1972 to 2008 using improved extraction methods[3] and sophisticated equipment.[4] Mass spectrometry was used for the first time in 1972 during the Munich Olympics. Since then various new chromatographic techniques viz. high-resolution mass spectrometry (Atlanta Olympics Games 1996), isotope ratio mass spectrometry (Special Olympic Winter Games, 1998) and liquid chromatography mass spectrometry (Athens Olympic Games, 2004) have come into dope testing. There are a total of 35 WADA accredited labs in the world. Each lab has their own set testing protocols utilizing various equipment viz. GC, GC/MS, HRMS, LC/MS/MS and IRMS. There are a number of methods available for individual groups of drugs viz. diuretics,[5] beta blockers,[6] stimulants and glucocorticosteroids[7] but during testing in our lab it was felt that apart from certain drugs like glucocorticosteroids for which it is mandatory to detect on LC/MS/MS, there are a few other drugs in various other categories of drugs viz. anabolic steroids (6), diuretics (1), stimulants (4) which are required to be included in the LC/MS/MS methods because of difficulty in detection by conventional GC and GC/MS. Therefore, the purpose of the present study was to explore the possibility of detecting by the glucocorticosteroids method a few other drugs which were difficult to detect at required lower concentration levels by GC and GC/MS methods.

## Materials and Methods

### Chemicals

All reference standards were purchased from Sigma (St. Louis, MO, USA), Steraloids (Newport, USA) or National Measurement Institute (Pymble, NSW, Australia). Acetonitrile and ethyl acetate were obtained from Qualigens (Worli, Mumbai, India), methanol from JT Baker (Phillipburg, USA), tertiary butyl methyl ether (TBME) from Acros Organics (New Jersey, USA), formic acid from Merck (Worli, Mumbai, India). De-ionized water was prepared on a MilliQ Laboratory Plant (Millipore, Bedford, USA). Organic solvents and reagents were all of analytical grade.

### LC and MSMS Apparatus

The chromatographic system consisted of an Agilent 1100 series (Agilent Technologies, Waldbronn, Germany) equipped with G1311A high pressure gradient pumping system, G1329A Autosampler, G1379A degasser, G1316A column compartment and G1315B diode array detector. Mass spectrometric analyses were conducted using an API 3200 Triple quadrupole instrument (Applied-Biosystem-Sciex, Concord, Canada), equipped with a pneumatically assisted APCI (heated nebulizer) and ESI (electrospray) ionization source. The main analytical parameters of the mass spectrometer are summarized in Table 1. The whole system was controlled using Analyst 1.4^®^ software (Applied-Biosystem-Sciex, Concord, Canada).

**Table 1:** Tandem mass snectrometer and HPLC wnrkinn Darameters

*Parameter*	*Value*
**Tandem mass spectrometer**	**working parameters**
Scan type	MRM
Polarity	Positive/Negative
Ion source	ESI
Nebulizing gas	3
Curtain gas	10
Source temperature	550oC
**HPLC working parameters**	
Column	C-18 ODS Inertsil, 50 × 4.6 × 3.5 μm
Flow	700 μl per minute
Injection volume	10 μl
Mobile phase	1% Formic acid, Acetonitrile
Gradient	0-5 min B 15%, 5-6 min B 60%, 6-7 min B 100%, 7-7.10 min B 15%, 7.10-11.0 min B 15%

### Sample Preparation Procedure

**Figure 1:**
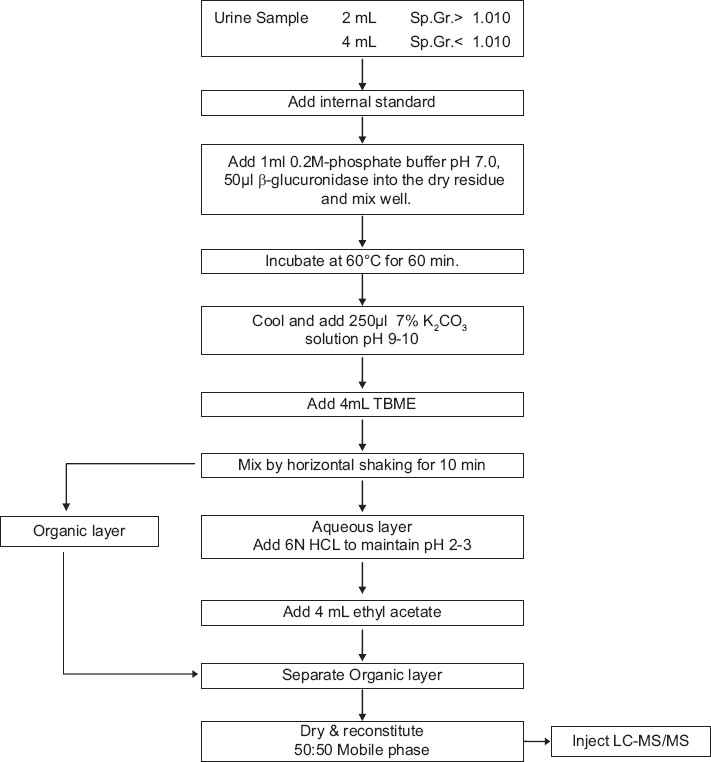
Sample preparation flow steps

A common procedure for the isolation of acidic, basic and neutral drugs from urine sample prior to LC/MS/MS was developed. The sample extraction procedure was illustrated in Figure 1. Table 2 reports different categories of drugs extracted by this procedure.

**Table 2:** Category of compounds analyzed

*Category*	*Compound*
Diuretic	Chlorothiazide
Stimulants	Ritalinic acid, Methyl Phenidate, Mesocarb
Anabolic androgenic steroids	Formebolone, Clomiphene metabolite, Epiternbolone, Gestrinone, Methy testosterone, THG, 3-OH-Stanozolol
Glucocorticosteroids	Triamcinolone, Prednisolone, Prednisone, Hydrocortisone, Fludrocortisone, Methyl prednisolone, Betamethasone, Dexamethasone, Flumethasone, Bedomethasone, Triamcinolone Acetonide, Desonide, Flunisolide, Fluocortolone, Fludrocortisone Acetate, Budesonide
5 α - reductase inhibitor	Carboxy finasteride

## Method development

### Electrospray ionization (ESI) and LC conditions

Mobile phase was introduced into the mass spectrometer via the ESI source operating in the positive ion/negative ion mode under multiple reaction monitoring conditions (MRM). Nitrogen was used as the nebulizing and curtain gas. Fragmentation was achieved with nitrogen. The dwell time for each transition was 60 msec and interchannel delay was 5 msec. For maximum sensitivity, the mass spectrometer parameters such as nebulizing gas, curtain gas and collision gas were optimized and collision energy is presented in Table 3. The temperature was 550°C and resolution was set at the unit.

**Table 3:** Analyte dependent mass spectrometry parameter for doping compounds

*Analyte*	*ESI lonization*	*Precursor Ions*	*Product Ions*	*Collision Energy*
Ritalinic acid	+ve	220	84	30
Chlorothiazide	−ve	294	214,179	−50
Methyl Phenidate	+ve	234	84	30
Triamcinolone	+ve	395	357,225	20
Formebolone	+ve	347	173	30
3-OH-Stanozolol	+ve	345	97,121,	30
			107,111,	
			219	
Mesocarb	+ve	339	193,135	30
Prednisolone	+ve	361	343,307	30
Prednisone	+ve	359	147,341	25
Hydrocortisone	+ve	363	121	31
Clomiphene metabolite	+ve	422	100,72	30
Fludrocortisone	+ve	381	181,105	35
Carboxy finasteride	+ve	403	335,175	30
Methyl prednisolone	+ve	375	161,357	30
Betamethasone	+ve	393	147,355	30
Dexamethasone	+ve	393	147,355	30
Flumethasone	+ve	411	253,121	26
Bedomethasone	+ve	409	279,391	30
Triamcinolone Acetonide	+ve	435	213,397	27
Desonide	+ve	417	399,147	27
Flunisolide	+ve	435	321,121	25
Fluocortolone	+ve	377	171,303	22
Epitrenbolone	+ve	271	199,227	30
Fludrocortisone Acetate	+ve	423	239,181	45
Budesonide	+ve	431	173,323	33
Gestrinone	+ve	309	241	30
Methy testosterone	+ve	303	109	30
THG	+ve	313	241	30

### LC conditions

**Figure 3:**
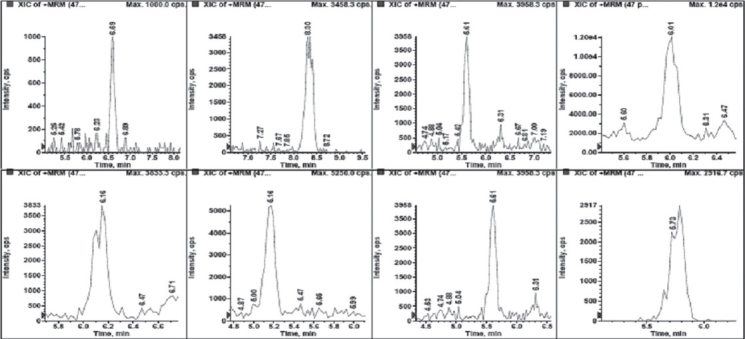
Single MRM transition of few doping drugs

The chromatogram was on Inertsil^®^ ODS-3 (3.0 um, 50 mm × 4.6 mm i.d.) C_18_ column (GL Sciences Inc., Tokyo, Japan) at 25°C temperature. Figure 3 shows the chromatogram of the single MRM transition of a few doping agents. The mobile phase composition was a mixture of 1% formic acid and acetonitrile with gradient time period. The flow rate was 700 *μ*l per minute.

## Results

### Internal standard

17-α methyl testosterone was used as the internal standard. The total recovery and elution time of the internal standard were perfectly fit for the chromatographic conditions of the method.

### Limit of detection (LOD)

Limit of detection is defined as the measure of the background noise with that of the baseline peak. Three times the standard deviation of noise in the negative urine was used to estimate the limit of detection. LOD was measured by spiking seven aliquots of negative urine samples. The analysis of these was done for a period of few weeks for instrument performance. LOD varied much for steroids, diuretics and glucocorticosteroids due to differences in the ionization criteria of individual drugs. Table 4 shows the LOD of different drugs.

**Table 4:** Compounds with limit of detection (LOD), retention time, linear ranqe and CV%

*Analyte*	*RT (Min)*	*LOD (ng/ml)*	*Linear range (ng/ml)*	*CV%*
Ritalinic acid	2.86	NA	NA	NA
Chlorothiazide	2.98	5	50-500	10.5
Methyl Phenidate	3.00	5	50-500	10.5
Triamcinolone	4.46	2	5-60	2.1
Formebolone	4.64	5	5-50	10.3
Mesocarb	4.68	NA	NA	NA
3-OH-Stanozolol	4.90	1	1-30	12.3
Prednisolone	5.09	2	15-120	10.2
Prednisone	5.10	2	15-120	6.3
Hydrocortisone	5.15	NA	NA	NA
Clomiphene metabolite	5.15	NA	NA	NA
Fludrocortisone	5.26	2	5-60	9.8
Carboxy finasteride	5.31	0.5	5-60	2.1
Methyl prednisolone	5.65	3	15-120	10.7
Betamethasone	5.77	1	1-20	13.6
Dexamethasone	5.77	1	1-20	13.6
Flumethasone	5.81	1	15-120	0.5
Beclomethasone	5.99	5	15-120	8.5
Triamcinolone Acetonide	6.12	2	15-120	4.7
Desonide	6.15	2	15-120	3.7
Flunisolide	6.15	1	15-120	11.5
Fluocortolone	6.34	1	15-120	5.4
Epiternbolone	6.45	2	5-60	8.6
Fludrocortisone Acetate	6.51	2	15-120	5.4
Budesonide	7.19	1	15-120	6.4
Gestrinone	7.30	3	5-50	4.3
Methy testosterone	7.45	NA	NA	NA
THG	8.35	1	2.5-20	7.1

### Calibration and quality control samples

Calibration standards were prepared by addition of ten microliters of working solution to drug free urine (DFU) to obtain required concentration levels. Quality control samples were prepared at concentrations 30 ng/ml (glucocorticosteroids), 10 ng/ml (steroids except 3-OH-stanozolol at 2 ng/ml) and 100 ng/ml (diuretics and stimulants) for minimum required performance limit (MRPL) set by WADA. The internal standard (17 α-Methyl testosterone) solution (500 ng/ml) was prepared by diluting its stock solution with methanol. Working solutions were prepared from the stock solution by dilution using methanol.

### Calibration curve

A five-point calibration curve was plotted for each drug with the internal standard covering the LOD and MRPL. The calibration curves were generated for three consecutive days and linearity was assessed [Table 4]. The calibration curve had a correlation coefficient of 0.9998. The five-point calibration curve gave acceptable results for the standard and was used for all quantitative calculations.

### Precision and accuracy

Precision and accuracy was determined by analyzing six sets of quality control samples. The within batch and between batch precision and accuracy was done by randomized daily process and analyzed in position at the end of the batch. Table 4 summarizes the cumulative variance of all drugs.

### Specificity and matrix effect

**Figure 2:**
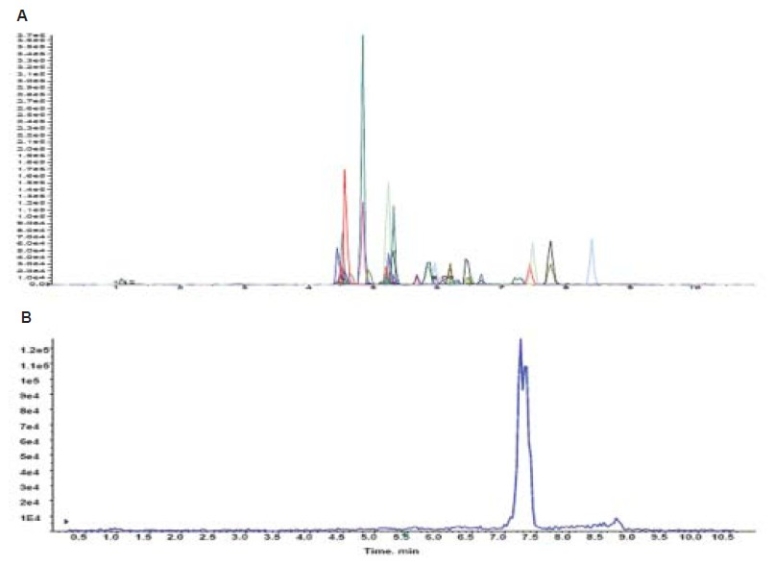
LCMSMS total ion chromatogram resulting from (A) analysis of urine sample spiked with mixture of doping agents; (B) blank urine sample with internal standard

The negative urine sample is spiked with the internal standard to find the specificity and matrix effect of the method. Figure 2(b) shows the total ion chromatogram of blank urine sample with internal standard. Matrix interferences were observed due to the short run time but eliminated by increasing post run time of the method. The method found to be specific for the drugs summarized in Figure 2(a).

### Total recovery

**Figure 4:**
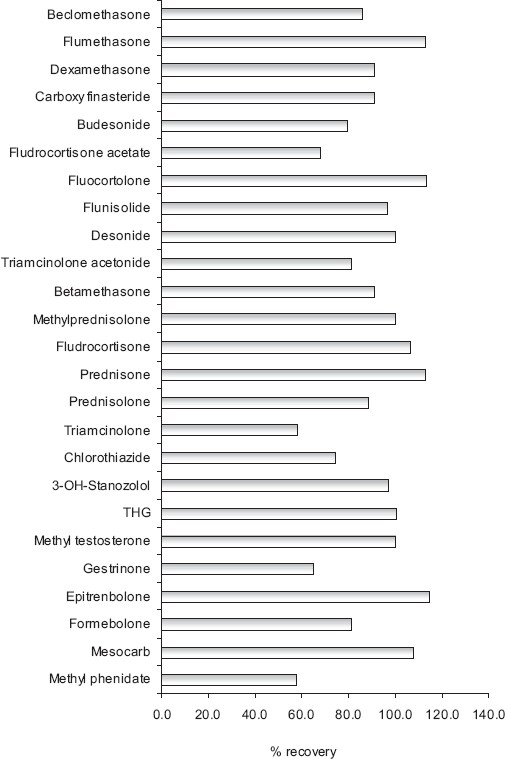
Average recoveries for extraction of doping agents

Total recovery completely relied on the sample preparation procedure. The total recovery of steroids, glucocorticosteroids, diuretics and narcotics was found to be good and acceptable with range of 57 to 114% as these compounds were extracted at different pH conditions. The total recovery was evaluated by comparing the mean peak areas of ten processed samples with the mean peak areas of unprocessed direct reference standard solutions of same concentrations. Figure 4 reports average recoveries for extraction of doping agents.

## Discussion

The present method developed on HPLC-mass spectrometer could detect all the 28 doping agents in a single run with the limit of detection set by WADA. The total run time of the present method was short with better separation. In the last 3 to 4 years, a number of methods have been published on analysis of doping agents on LC/MS/MS. However, these methods are on specific group of drugs viz. diuretics,[5] beta-blockers,[6] glucocorticosteroids[7] and a few anabolic steroids.[8] The present method was developed for 14 glucocorticosteroids and 6 anabolic steroids and then extended to a few estrogenic drugs, stimulants and diuretics, thereby detecting a total of 28 drugs in a single screen. It helped to detect problematic drugs from diuretics (chlorothiazide), stimulants (methyl phenidate, mesocarb) and steroids (3-OH-stanozolol) thereby making one versatile method for various drugs. The ionization and fragmentation pattern differs for each category of drugs; the results suggest that glucocorticosteroids are neutral compounds to ionize[9] whereas steroids and diuretics are polar[10] and easily fragmentable. Also observed was no polarity switching between chlorothiazide (detected at a retention time of 2.98 min in negative mode) and methyl phenidate (detected at a retention time of 3.00 min in positive mode) though they are co-eluting substances. Furthermore, analysis shows stimulants and narcotics are highly sensitive and ESI appeared to be the best sensitive ionization for methyl phenidate. The ionization pattern and separation were earlier discussed in a few publications which suggest buffering agents are significant in ionization.[11] In the present study formic acid and acetonitrile are used as eluting mobile phases that gave good separation and sensitivity. The method was successfully used to detect positive urine samples of 3-OH-stanozolol, methylphenidate, mesocarb, clomiphene metabolite and carboxy finasteride. The positive urine samples were collected for 48-72 hrs after single dose administration of 3-OH-stanozolol, methyl phenidate, mesocarb, clomiphene and finasteride to healthy volunteers. The drugs and metabolites could be detected up to 48 hrs later. However, methyl phenidate and ritalinic acid were tested in a positive sample purchased from Australian Doping Lab. The total recovery percentage ranged from 57 to 114 where the lower end of the recovery may be due to the matrix associated with the samples at different pH conditions. Further, the recovery percentage for different categories of drugs done by this method is at concentrations of 10 ng/ml for steroids except 3-OH-Stanozolol at 2 ng/ml, 250 ng/ml for stimulants, 100 ng/ml for diuretics, 30 ng/ml for glucocorticosteroids and 100 ng/ml for carboxy finasteride. The extraction recovery percentage in the range of 80-120% is considered to be acceptable by the international validation protocols.[12] However, the protocol for bioanalytical method validation explains that the recovery of an analyte need not be 100%, but the extent of recovery of an analyte and of the internal standard should be consistent, precise and reproducible.[13] The validated method was used for testing of 1000 In-competition samples successfully. However, during this testing adverse analytical findings were reported for 3-OH-Stanozolol in ten samples.

**Figure 5:**
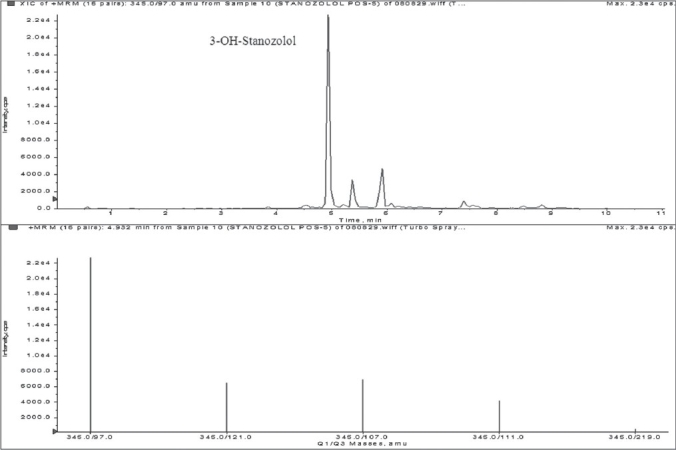
TIC and mass spectrum of 3-OH-Stanozolol from human urine excretion study sample

In conclusion, the method developed based on controlled pH extraction method and HPLC-mass spectrometry analysis allowed better identification and confirmation of chemically and pharmacologically different banned drugs in urine in a single short run. The method could detect target analytes at a concentration of minimum required performance limit set by WADA. The assay provides a useful alternative approach to individual methods for screening and confirmation of a few diuretics, stimulants, narcotics, steroids and glucocorticosteroids. The approach has great potential in doping and clinical testing and might simplify the analytical screening and confirmation cost effectively.
